# Supplementary material for: The causal role of circulating inflammatory markers in osteoporosis: a bidirectional Mendelian randomized study
Source: Front Immunol. 2024 Jul 18;15:1412298. doi: 10.3389/fimmu.2024.1412298 (PMC11291241; doi:10.3389/fimmu.2024.1412298)
Supplement: Supplementary file 1 [file DataSheet_1.zip › supplementary material/OSTEOPOROSIS-CIM/GCST90274802.tsv.gz_forest.pdf]

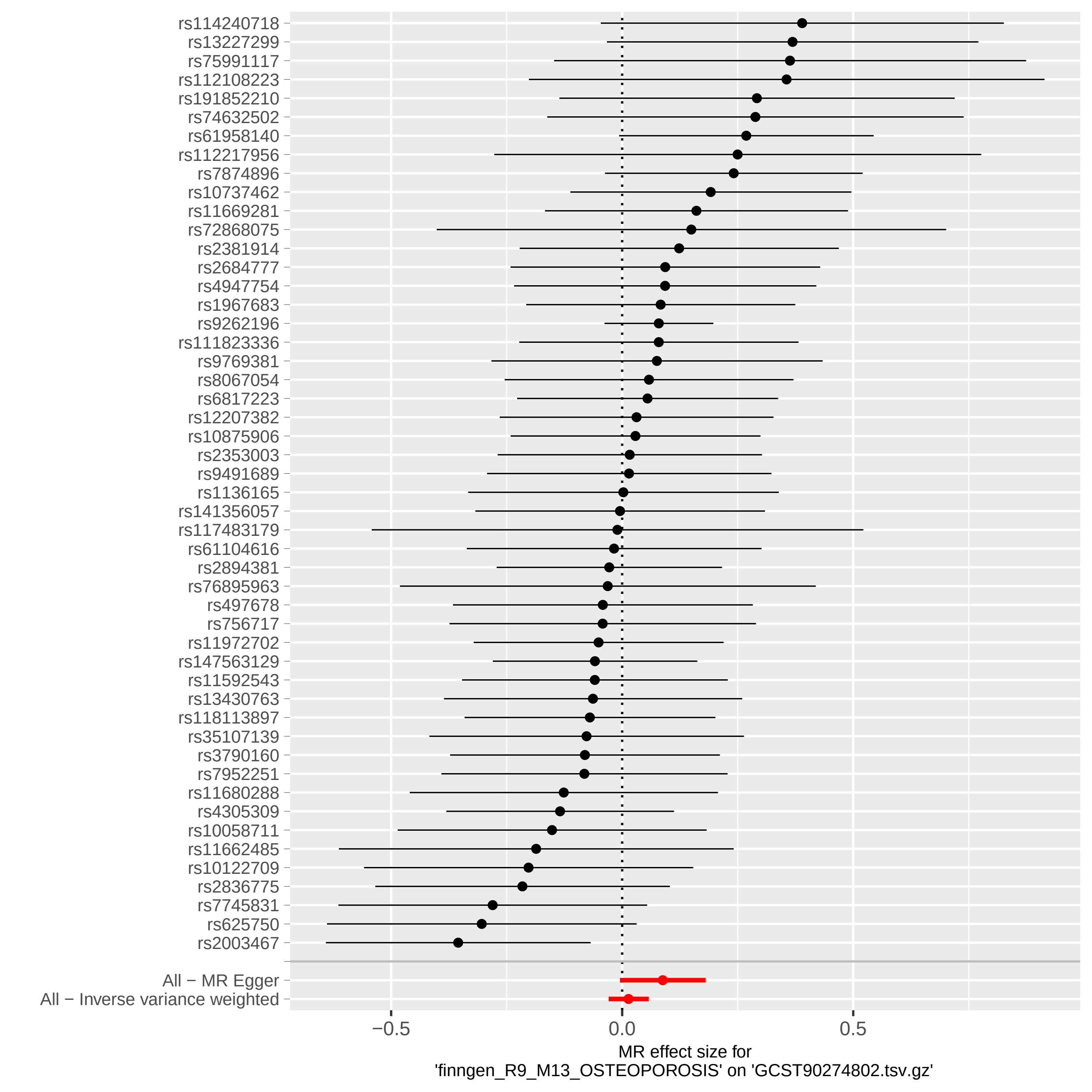
rs114240718
rs13227299
rs75991117
rs112108223
rs191852210
rs74632502
rs61958140
rs112217956
rs7874896
rs10737462
rs11669281
rs72868075
rs2381914
rs2684777
rs4947754
rs1967683
rs9262196
rs111823336
rs9769381
rs8067054
rs6817223
rs12207382
rs10875906
rs2353003
rs9491689
rs1136165
rs141356057
rs117483179
rs61104616
rs2894381
rs76895963
rs497678
rs756717
rs11972702
rs147563129
rs11592543
rs13430763
rs118113897
rs35107139
rs3790160
rs7952251
rs11680288
rs4305309
rs10058711
rs11662485
rs10122709
rs2836775
rs7745831
rs625750
rs2003467
All − MR Egger
All − Inverse variance weighted
−0.5
0.0
0.5
MR effect size for
'finngen_R9_M13_OSTEOPOROSIS' on 'GCST90274802.tsv.gz'
